# Traumatic Stress Produces Distinct Activations of GABAergic and Glutamatergic Neurons in Amygdala

**DOI:** 10.3389/fnins.2018.00387

**Published:** 2018-08-21

**Authors:** Qing Fang, Zhe Li, Geng-Di Huang, Huan-Huan Zhang, Ya-Yun Chen, Li-Bo Zhang, Zeng-Bo Ding, Jie Shi, Lin Lu, Jian-Li Yang

**Affiliations:** ^1^Department of Psychiatry, Tianjin Medical University, Tianjin, China; ^2^National Institute on Drug Dependence and Beijing Key Laboratory of Drug Dependence, Peking University, Beijing, China; ^3^Psychiatric Department, Tianjin Anding Hospital, Tianjin, China; ^4^Cangzhou Medical College, Cangzhou, China; ^5^Peking University Sixth Hospital/Peking University Institute of Mental Health, Peking University, Beijing, China; ^6^Tianjin Medical University General Hospital, Tianjin, China

**Keywords:** posttraumatic stress disorder (PTSD), single prolonged stress (SPS), basolateral amygdala (BLA), central amygdala (CeA), neuronal activations

## Abstract

Posttraumatic stress disorder (PTSD) is an anxiety disorder characterized by intrusive recollections of a severe traumatic event and hyperarousal following exposure to the event. Human and animal studies have shown that the change of amygdala activity after traumatic stress may contribute to occurrences of some symptoms or behaviors of the patients or animals with PTSD. However, it is still unknown how the neuronal activation of different sub-regions in amygdala changes during the development of PTSD. In the present study, we used single prolonged stress (SPS) procedure to obtain the animal model of PTSD, and found that 1 day after SPS, there were normal anxiety behavior and extinction of fear memory in rats which were accompanied by a reduced proportion of activated glutamatergic neurons and increased proportion of activated GABAergic neurons in basolateral amygdala (BLA). About 10 days after SPS, we observed enhanced anxiety and impaired extinction of fear memory with increased activated both glutamatergic and GABAergic neurons in BLA and increased activated GABAergic neurons in central amygdala (CeA). These results indicate that during early and late phase after traumatic stress, distinct patterns of activation of glutamatergic neurons and GABAergic neurons are displayed in amygdala, which may be implicated in the development of PTSD.

## Introduction

Posttraumatic stress disorder (PTSD) is a complex, chronic mental disorder, and its lifetime prevalence is approximately 1.0–2.6% in the general population (APA, 2000, 2013b). The vulnerable individuals are susceptible to the stress events of life-threatening illness or serious injuries such as traffic accidents, sexual assault, rape, natural disasters, or wars (Olaya et al., 2015; Benjet et al., 2016). This mental disorder contains three main symptom clusters: the re-experiencing and intrusive memories of the traumatic event (TE), avoidance behaviors, and emotional numbing, a sustained state of hypervigilance and startle response enhancement, which finally affect patients’ daily skills (APA, 2000, 2013a). At present, there has been great progress in the pharmacotherapy of PTSD. Some drugs, for example, selective serotonin reuptake inhibitors, have been used for the first-line treatments for PTSD (Ipser et al., 2006; Corchs et al., 2009). Nevertheless, there is still a subgroup of PTSD patients who have some persistent symptoms or maintain conditioned fear responses to TEs, finally being chronic.

Scientists have conducted many preclinical animal paradigms in an attempt to model PTSD for better understanding the pathophysiology of this disorder in the past few decades. Substantial evidence has supported the face and constructs validity of the single prolonged stress (SPS) model. SPS comprises a SPS episode involving serial exposures to multiple stressors (restraint, forced swim, ether), followed by a “no-touch” quiescent period of 7 days (Liberzon et al., 1997, 1999; Yamamoto et al., 2009). A quiescent period of time is required for the SPS rats to exhibit behavioral characteristics of PTSD, including delayed occurrence of anxiety-like behaviors, startle response enhancement and impairment of fear extinction (Khan and Liberzon, 2004; Wen et al., 2008; Knox et al., 2012). SPS also induces a few pathophysiological characteristics of PTSD, for example, the representative abnormalities of hypothalamic-pituitary-adrenal (HPA) axis (Liberzon et al., 1997).

The amygdala is located deep and medially within the temporal lobes of the brain, and is considered part of the limbic system. Its pivotal components include the basolateral amygdala (BLA) and the central nucleus (CeA). The BLA is cortex-like with a dominant group of glutamatergic projection neurons (∼80%) and a minority of local-circuit GABAergic interneurons (∼20%) (Duvarci and Pare, 2014). However, the CeA is similar to the striatum (Ehrlich et al., 2009), predominantly composed of medium-size spiny, GABAergic inhibitory neurons (∼95%) (Mcdonald, 1982; Cassell et al., 1986; Swanson and Petrovich, 1998). In recent years, accumulating studies have focused on brain structures involved in the pathophysiology of PTSD, including amygdala (Liberzon and Sripada, 2008; George et al., 2013; Kwapis and Wood, 2014). Human studies suggested a hyper-responsive activity including the amygdala in PTSD. Exaggerated of the amygdala activity has been observed in response to trauma-related stimuli (Shin et al., 1997; Liberzon et al., 1999; Pissiota et al., 2002; Hendler et al., 2003), and also during acquisition of conditioned fear (Bremner et al., 2005). Using animal model of PTSD, a few studies found that 5-HT2C (Harada et al., 2008), Rin1 and Stathmin (Han et al., 2017), beta-adrenoreceptors (Ronzoni et al., 2016), Neuropeptide S (Cohen et al., 2018), and beta-arrestin-2 (Ding et al., 2017) may be implicated in PTSD associated behaviors. Recently, through using c-Fos or other markers of neuronal activity, increased activation of the amygdala was found to be associated with traumatic stress-induced behavioral changes, such as anxiety-like behaviors and deficit in fear extinction (Muigg et al., 2008; Hoffman et al., 2014; Ritov et al., 2014; Yu et al., 2015; Knox et al., 2018). However, the temporal (early versus late phase after stress) and neuron-subtype (excitatory versus inhibitory activity) changes after traumatic stress are still unknown.

Brain function depends on the dynamic balance of excitatory/inhibitory neural networks to maintain steady activity and keep the response normal. As the primary excitatory neurotransmitter in the central nervous system, glutamate acts through ionotropic receptors and metabotropic glutamate receptors (Chambers et al., 1999), and is involved in rapid information transmission between the cortex, hippocampus, and amygdala structures. An increase in glutamate transmission occurred across different brain regions, such as the amygdala and hippocampus, of rats after acute stress exposure (Pitman et al., 2012). GABAergic inhibitory control is pivotal for the precise regulation of expression, consolidation and extinction of fear conditioning (Zhang and Cranney, 2008; Bolshakov, 2009; Makkar et al., 2010). The GABAergic signaling of BLA was proved to be decreased in fear conditioning compared with that of unconditioned controls (Rea et al., 2009). In human studies, numerous magnetic resonance spectroscopy studies have indicated abnormal glutamate and GABA levels in the brain of PTSD patients (Averill et al., 2016). Consistently, the clinical trials have showed that GABAergic drugs can enhance the neuronal activity by combining the GABAergic receptors, and the patients who take regular medication of these drugs could decrease the anxiety-like behaviors (Harpaz-Rotem et al., 2008; Lund et al., 2013). In animal studies, SPS procedures decreased glutamate, glutamine, and creatine levels (Knox et al., 2010). D-cycloserine (DCS), one kind of partial N-methyl-D-aspartate (NMDA) receptor agonists, has been shown to facilitate extinction of learned fear (Ledgerwood et al., 2005; Guastella et al., 2007; Otto et al., 2010; Mccallum et al., 2011), and prevented SPS-induced changes in fear extinction and hippocampal NMDA receptor mRNA expression (Yamamoto et al., 2008). Taken together, a handful of studies have shown changes of glutamatergic and GABAergic transmission after occurrence of PTSD symptoms in animal models and patients. However, it is still not clear how GABAergic and glutamatergic networks change over time during the development of PTSD-like behaviors. Thus, in the present study, we attempt to reveal the role of pathological alterations of glutamatergic and GABAergic activation in the development of PTSD-like behaviors using the SPS animal model.

## Materials and Methods

### Subjects

All of the experiments were approved by the National Institutes of Health Guide for the Care and Use of Laboratory Animals and Biomedical Ethics Committee of Peking University for animal use and protection. The protocol was approved by the Biomedical Ethics Committee of Peking University for animal use and protection. Male Sprague–Dawley rats (3-month-old, 200–220 g) were obtained from Beijing Vital River Laboratory Animal Technology Co., Ltd. These rats were housed five per cage with *ad libitum* access to food and water under a 12-h/12-h light/dark cycle (lights on at 08:00 P.M.). After 5 days’ habitation, some rats were used for PTSD model through the SPS protocol, and then both the SPS groups and the control ones are kept in the normal breeding cages separately. The behavioral experiments were conducted during the dark phase of the cycle.

### SPS Procedure

The SPS procedure was based on previous studies (Ding et al., 2010). Rats were placed into a plastic restrainer which is close enough to the body but allows the normal breathing for 2 h. Then, the subjects were immediately putted into the water (40-cm depth, temperature 25°C) for a 20-min forced swim. After a 15-min rest, the rats were given a loss of consciousness by the diethyl anesthetization.

### Open Field Test (OFT)

The OFT was based on our previous studies (Xue et al., 2015), and was conducted in a procedure room under dim light conditions. The test was recorded for 5 min per rat and then analyzed with an EthoVision System XT 10.1 (Noldus Information Technology, Netherlands). In the OFT test, the total travel distance and the time spent in the central area were counted to measurement the rats’ behavior.

### Elevated Plus Maze (EPM)

The elevated plus maze (EPM) test was based on our previous studies (Xue et al., 2015), and the test time and condition is similar to the OFT. EPM was an apparatus with two open arms and two closed arms (each: 10-cm width^∗^50-cm length^∗^70-cm height; the closed arms: 40-cm depth). The apparatus was cleaned by the 75% ethanol-soaked towel to remove the odor of the previous animal. Then, we would wait for a few minutes to ensure the apparatus was dry and the smell of ethanol in the test box was mild. Each rat was allowed to explore the arena freely and recorded for 5 min, then analyzed with an EthoVision System XT 10.1 (Noldus Information Technology, Netherlands). In the data analysis, the number of arm entries and the proportion of time spent in the open arms [time in open arms/(open + closed arm)] time were calculated.

### Contextual Fear Conditioning (CFC) and Extinction

The CFC procedure was based on our previous studies (Liu et al., 2015; Xue et al., 2015). The rats were handled for 3 days before conditioning. On the day of the experiment, they were placed in the conditioning chamber (Beijing Macro Ambition S&T Development Co., Ltd., Beijing, China) and allowed to explore the chamber for 2 min, after which they received an electric footshock (0.75 mA, 1 s). The 2 min/1 s procedure was repeated a total of three times. About 1 day after the CFC, the rats were exposed to the training chamber for 30 min without any footshock for extinction. Extinction retention test was performed 1 day later, during which rats were placed into the chamber for 5 min without footshock. In the CFC, we captured the freezing behavior and conditioned fear was assessed by measuring the percentage of time spent freezing during the 20-s period of each CS presentation. Freezing behavior was defined as the lack of all movement, with the exception of respiration. All sessions of the experiment were video-recorded by an animal behavior video analysis system (Beijing Macro Ambition S&T Development, Beijing, China) for offline analysis.

### Tissue Sample Preparation

On the day 1 or 10 after SPS, the rats were perfused at 90 min after the behavioral tests. The subjects were anesthetized with 4% pentobarbital sodium, perfused with 0.01 M PBS, and fixed by 4% paraformaldehyde (PFA), pH 7.40. Then, the brains were extracted and removed in 4% PFA for post-fixation. Subsequently, the brains were fast frozen and coronally sectioned at 20 μm, and the slices were used to the fluorescence immunohistochemistry.

### Fluorescence Immunohistochemistry

Fluorescence immunohistochemistry was performed per previous study (Xue et al., 2017) with some modifications. First, the brain slices prepared above were blocked with 2% bovine serum albumin (BSA) in the 37.0°C thermostat container for 1 h. Second, half of the specimens were incubated with rabbit primary antibody directed against the c-fos (1:500, Cell Signaling Technology, # 2250S) and mouse primary antibody directed against the GAD67 (1:500, Novus Biologicals, # MAB5406) on the 4°C shaker for 30 h; the incubation condition of other half specimens was the same, but the primary antibodies were rabbit anti-fos and mouse anti-CaMKIIα (1:500, Novus Biologicals, # NB100-1983). Third, the specimens were washed forth (^∗^5 min) in 0.01 M PBS solution (pH 7.40), then incubated with the second antibodies (Alexa Fluor 488 goat anti-rabbit IgG, 1:500, Thermo Fisher Scientific, # A11034; Alexa Fluor 594 donkey anti-mouse IgG, 1:500, Thermo Fisher Scientific, # A21203) on a shaker in the room temperature for 3 h. Lastly, the slices were washed forth again (^∗^5 min) in the 0.01 M PBS, and mounted on glass slides, then covered by coverslip. The slides were examined using a fluorescence microscopy (Olympus VS-120 microscopy) with a 20X objective lens.

### Statistical Analysis

The results were expressed as mean ± SEM. Shapiro-Wilks test was used to verify normal distribution and Levene’s test to verify homogeneity of variance. The data were analyzed using analysis of variance (ANOVA) with appropriate between- and within-subject factors for each experiment (see the Section “Results”). Significant main effects and interactions (*p* < 0.05, two-tailed) in the factorial ANOVAs were followed by one-way ANOVAs and the Least Significant Difference *post hoc* test.

## Results

### Experiment 1: Effect of Single Prolonged Stress (SPS) on Anxiety-Like Behaviors and Fear Extinction on Day 1 or Day 10 After Stress

**FIGURE 1:**
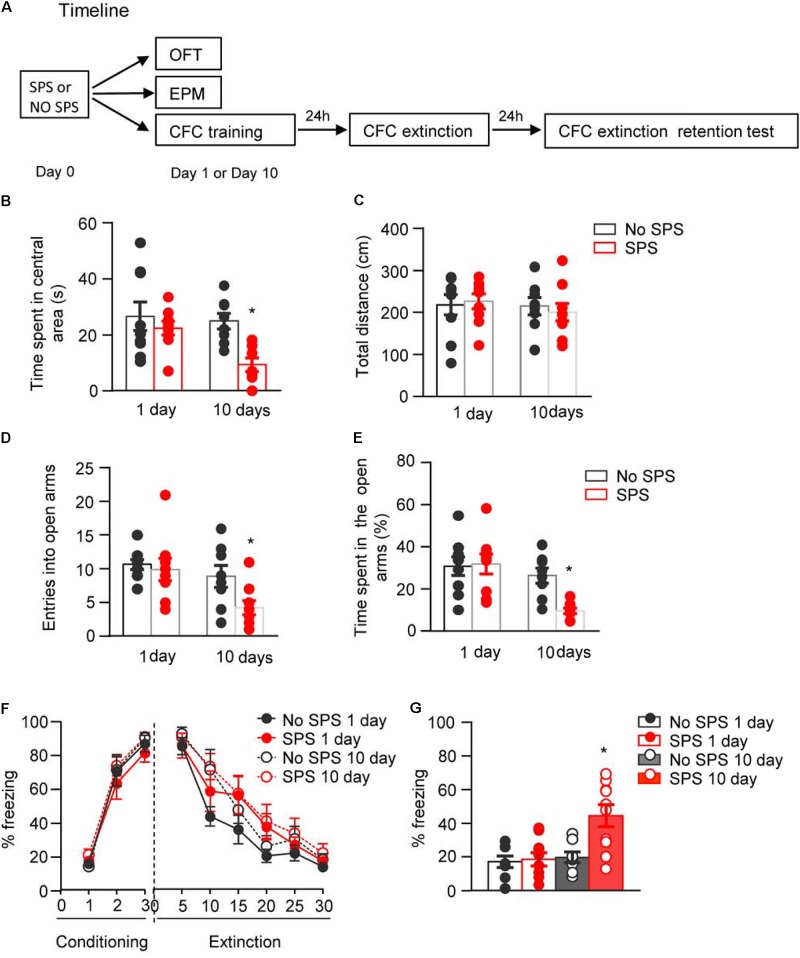
Effect of single prolonged stress (SPS) on anxiety-like behaviors and fear extinction on day 1 or day 10 after stress. **(A)** Experimental procedures. We treated rats with SPS procedure. About 1 day or 10 days later, we examined the anxiety-like behaviors with open field and elevated plus maze, and examined fear extinction with contextual fear conditioning. **(B,C)** Time spent in the central area **(B)** and the total locomotor distance **(C)** in different experimental conditions in open field test. **(D,E)** The entries into open arms **(D)** and time spent in open arms **(E)** in different experimental conditions in elevated plus maze test. **(F,G)** Formation and extinction of fear conditioning **(F)** and memory retention of fear extinction **(G)** in different experimental conditions. *n* = 8–10 per experimental condition. Data are mean ± SEM^2^. ^∗^Different from all other groups, one-way ANOVA, *p* < 0.05.

Previous studies have shown that enhancement of fast negative feedback of the HPA axis and alterations of neurotransmission in several brain regions including amygdala are observed up to 14 days after SPS (Liberzon et al., 1997, 1999; Knox et al., 2010). Thus, the behavioral test and molecular assay were conducted on day 1 and day 10 after SPS to examine the correlation relationship between the occurrence of PTSD associated behaviors (anxiety and impairment of fear extinction) and the neuronal activation patterns of amygdala. OFT and EPM were used to assess the SPS-induced anxiety-like behaviors, and CFC was applied to assess the SPS-induced impairment of fear extinction retention. In each behavioral test, rats were divided into four groups, and two groups of rats were exposed to SPS while another two groups were kept in the home cage as the control. Different cohorts of rats received behavioral test on day 1 or day 10 after SPS, respectively (**Figure 1A**).

First, four groups of rats (*n* = 8–9 per group) were used to examine the behavioral alterations in OFT at different time points after SPS procedure. Two-way ANOVA, with Stress (SPS, No SPS) and Post-stress Day (1 day, 10 days) as the between-subject factors, was used to analyze the behavioral data of time spent in the central area and total locomotor distance. For time spent in the central area, it revealed significant effects of Stress (*F*_1,31_ = 8.39, *p* < 0.01) and Post-stress Day (*F*_1,31_ = 4.63, *p* < 0.05). *Post hoc* analysis revealed that compared with other groups, SPS-10 days group showed a significant decrease in the time spent in the central area (*p* < 0.05, **Figure 1B**). For total locomotor distance, there were no significant differences among experimental conditions (*p* > 0.05, **Figure 1C**).

Then another four groups of rats (*n* = 8–9 per group) were used to examine the behavioral alterations in EPM at different time points after SPS procedure. The data analysis methods were the same as OFT experiment. The analysis of behavioral data of entries into open arms and time spent in open arms revealed significant effects of Stress (*F*_1,31_ = 4.25, *p* < 0.05; *F*_1,31_ = 4.36, *p* < 0.05, respectively) and Post-stress Day (*F*_1,31_ = 8.02, *p* < 0.01; *F*_1,31_ = 12.60, *p* < 0.01, respectively). *Post hoc* analysis revealed that compared with other groups, SPS-10 days group showed a significant decrease in the percentage of entries into open arms (**Figure 1D**) and time spent in the open arms (both *p* < 0.05, **Figure 1E**).

Another four groups of rats (*n* = 8–10 per group) were used to examine the effect of traumatic stress on fear extinction at different time points after SPS. Two-way ANOVA, with Stress (SPS, No SPS) and Post-stress Day (1 day, 10 days) as the between-subject factors and Extinction Session or Retention Test as the within-subject factor, was used to analyze the behavioral data of fear formation, fear extinction and memory retention of fear extinction. The analysis of behavioral data of fear formation and fear extinction revealed only a significant effect of Session (*F*_2,64_ = 209.91, *p* < 0.01 for Conditioning Session; *F*_5,160_ = 73.32, *p* < 0.01 Extinction Session, **Figure 1F**). The analysis of behavioral data of fear extinction retention revealed a significant effect of Retention Test (*F*_1,32_ = 11.67, *p* < 0.01) and interactions of Retention Test × Stress (*F*_1,32_ = 4.24, *p* < 0.05) and Retention Test × Post-stress Day (*F*_1,32_ = 4.53, *p* < 0.05). *Post hoc* analysis revealed that SPS-10 days group showed a significant increase of freezing in extinction retention test (both *p* < 0.05, **Figure 1G**) of the SPS-10 days compared with other groups.

These results indicate that SPS induces anxiety-like behavior and impairment of extinction retention on day 10 but not day 1 after stress. This finding is consistent with previous studies that there is a delayed occurrence of PTSD-associated behaviors (Knox et al., 2012; Wu et al., 2016).

### Experiment 2: Effect of SPS on Activations of Glutamatergic and GABAergic Neurons of BLA and CeA on Day 1 or Day 10 After Stress

**FIGURE 2:**
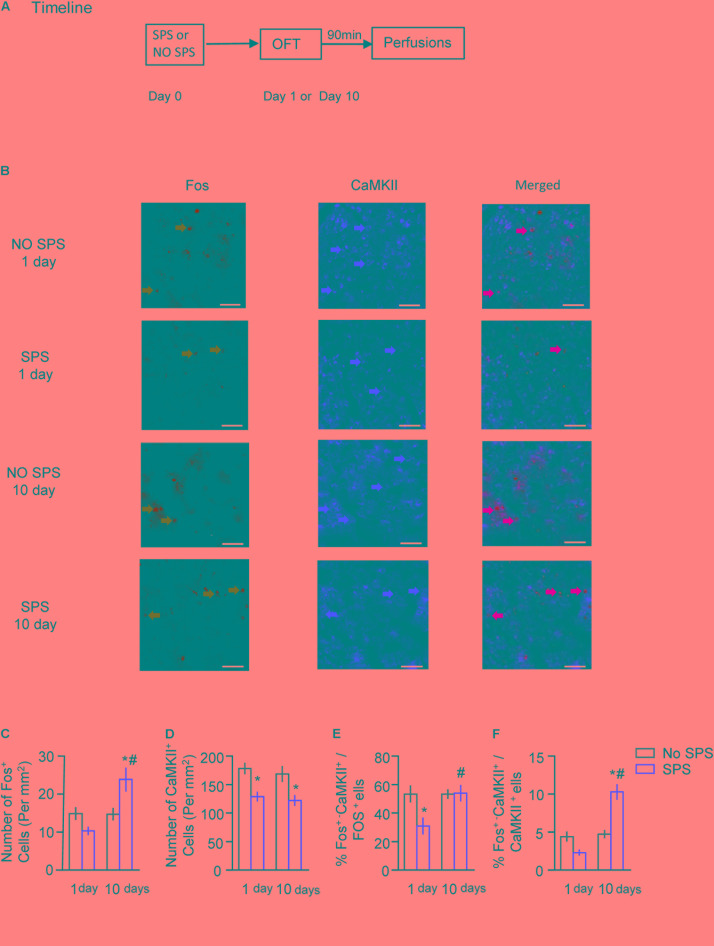
Effect of single prolonged stress (SPS) on activation of glutamatergic neurons in BLA on day 1 or day 10 after stress. **(A)** Experimental procedures. We treated rats with SPS procedure, and performed perfusion and brain dissection 1 day or 10 days later. **(B)** Representative images showing **green** (Fos protein), **red** (CaMKII protein), and merged channels of double-label neurons in BLA in different experimental conditions. Scale bars represent 100 μm. **(C,D)** Effect of the experimental manipulations on Fos protein **(C)** and CaMKII expression **(D)** in BLA. **(E)** Percentage of glutamatergic neurons in activated cells of BLA in different experimental manipulations, as calculated by number of overlap (Fos + CaMKII protein-IR)/Fos-IR. **(F)** Percentage of activated cells in glutamatergic neurons of BLA in different experimental manipulations, as calculated by number of overlap (Fos + CaMKII protein-IR)/CaMKII-IR. *n* = 6–8 per group. Data are mean ± SEM. ^∗^Different from no SPS group at each post-stress day. ^#^Different from SPS-1 day group, one-way ANOVA, *p* < 0.05.

CaMKII is selectively expressed in the excitatory glutamatergic neurons (Benson et al., 1992; Liu and Jones, 1996) and is involved in regulating the maturation and plasticity of glutamatergic synapses (Silva et al., 1992; Mayford et al., 1995; Wu et al., 1996; Rongo and Kaplan, 1999). On the other hand, glutamic acid decarboxylase (GAD) is the synthetic enzyme for GABA and is selectively expressed in GABAergic neurons (Gonzales et al., 1991). Therefore, we used the immunofluorescence methods to examine the levels of CaMKII and GAD67 and the numbers of CaMKII or GAD67 positive nuclei to represent the numbers of glutamatergic or GABAergic neurons, respectively. We investigated the activation pattern of glutamatergic and GABAergic neurons in BLA and CeA at different times after traumatic stress. Considering the neuronal composition in BLA and CeA (Swanson and Petrovich, 1998), we assessed the alterations of glutamatergic and GABAergic activity in BLA, and GABAergic activity in CeA. Rats were divided into four groups (*n* = 6–8 per group), i.e., SPS-1 day, No SPS-1 day, SPS-10 days, and No SPS-10 days. On day 1 or day 10 after SPS, rats were exposed to an open field (Hale et al., 2008) and perfused 90 min later. The expression of Fos, CaMKII and GAD67 were performed using fluorescence immunohistochemistry, and overlaps of the Fos&GAD67 and Fos&CamkII were used to reveal the activity of the glutamatergic and GABAergic neurons (**Figure 2A**), respectively. Two-way ANOVA, with Stress (SPS, No SPS) and Post-stress Day (1 day, 10 days) as the between-subject factors, was used to analyze the molecular data of numbers of Fos^+^, CaMKII^+^, and GAD67^+^ cells, as well as the percentages of Fos^+^-CaMKII^+^/Fos^+^, Fos^+^-CaMKII^+^/CaMKII^+^, Fos^+^-GAD67^+^/Fos^+^, and Fos^+^-GAD67^+^/GAD67^+^.

For immunofluorescence data of Fos and CaMKII in BLA (**Figure 2B**), the analysis revealed significant interaction of Stress × Post-stress Day (*F*_1,24_ = 11.12, *p* < 0.01 for number of Fos^+^ cells; *F*_1,24_ = 4.64, *p* < 0.05 for percentage of Fos^+^-CaMKII^+^/Fos^+^; *F*_1,24_ = 29.70, *p* < 0.01 for percentage of Fos^+^-CaMKII^+^/CaMKII^+^), and a significant effect of Stress on number of CaMKII^+^ cells (*F*_1,24_ = 19.88, *p* < 0.01). *Post hoc* analysis revealed a significant increase of number of Fos^+^ cells and percentage of Fos^+^-CaMKII^+^/CaMKII^+^ on day 10 (both *p* < 0.05, **Figures 2C,F**), but a significant decrease of number of CaMKII^+^ cells on both day 1 and 10 (both *p* < 0.05, **Figure 2D**). In addition, the decrease of number of CaMKII^+^ cells on day 1 returned to baseline on day 10 (both *p* < 0.05, **Figure 2E**). These results suggested on day 1 after stress, the number of CaMKII^+^ type cells was significantly decreased and proportion of activated cells in the CaMKII^+^ type cells was slightly decreased. Meanwhile, total number of activated cells was slightly decreased and the proportion of CaMKII^+^ type cells in the activated cells was significantly decreased. On day 10 after stress, the number of CaMKII^+^ type cells was still decreased while proportion of activated cells in the CaMKII^+^ type cells increased. Meanwhile, total number of activated cells was significantly increased and the proportion of CaMKII^+^ type cells in the activated neurons returned to the control levels in BLA.

**FIGURE 3:**
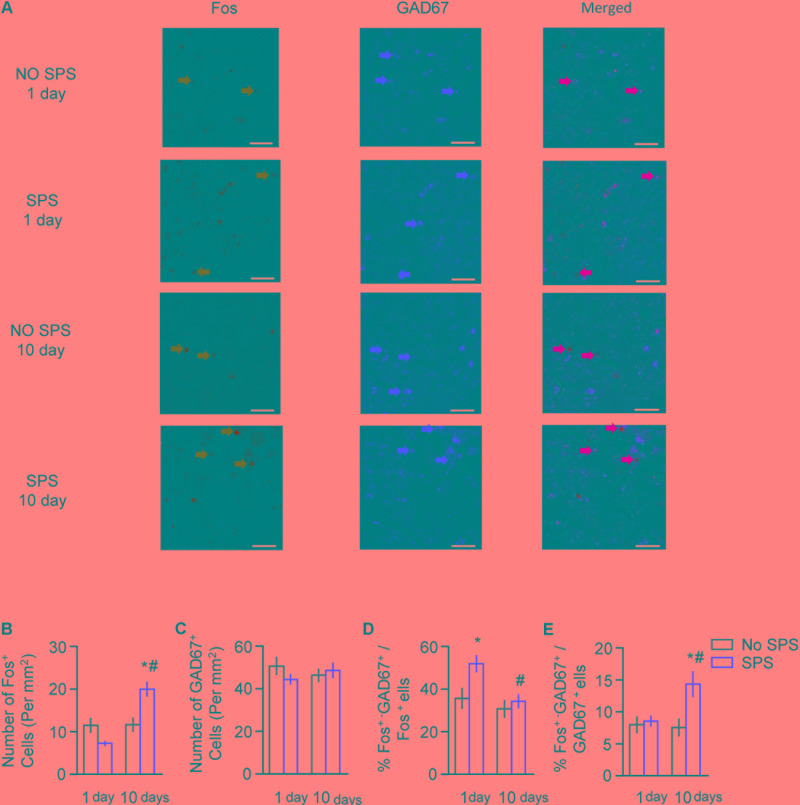
Effect of single prolonged stress (SPS) on activation of GABAergic neurons in BLA on day 1 or day 10 after stress. **(A)** Representative images showing **green** (Fos protein), **red** GAD67 protein), and merged channels of double-label neurons in BLA in different experimental conditions. Scale bars represent 100 μm. **(B,C)** Effect of the experimental manipulations on Fos protein **(B)** and GAD67 expression **(C)** in BLA. **(D)** Percentage of GABAergic neurons in activated cells of BLA in different experimental manipulations, as calculated by number of overlap (Fos + GAD67 protein-IR)/Fos-IR. **(E)** Percentage of activated cells in GABAergic neurons in different experimental manipulations, as calculated by number of overlap (Fos + GAD67 protein-IR)/GAD67-IR. *n* = 6–7 per group. Data are mean ± SEM. ^∗^Different from no SPS group at each post-stress day. ^#^Different from SPS-1 day group, one-way ANOVA, *p* < 0.05.

For immunofluorescence data of Fos and GAD67 in BLA (**Figure 3A**), the analysis revealed significant interaction of Stress × Post-stress Day (*F*_1,21_ = 17.18, *p* < 0.01 for number of Fos^+^ cells; *F*_1,21_ = 4.68, *p* < 0.05 for percentage of Fos^+^-GAD67^+^/GAD67^+^), and significant effects of Stress (*F*_1,21_ = 5.69, *p* < 0.05) and Post-stress Day (*F*_1,21_ = 7.39, *p* < 0.05) on percentage of Fos^+^-GAD67^+^/Fos^+^. *Post hoc* analysis revealed a significant increase of Fos^+^ cells and Fos^+^- GAD67^+^/GAD67^+^ on day 10 (both *p* < 0.05, **Figures 3B,E**), and a significant increase in percentage of Fos^+^-GAD67^+^/Fos^+^ on day 1 which returned to baseline on day 10 (both *p* < 0.05, **Figure 3D**). There are no significant changes of the total number of the GAD67 cells after stress on day 1 and day 10 (*p* > 0.05, **Figure 3C**). These results suggested that total number of activated cells was slightly decreased but the proportion of GAD67^+^ type cells in the total activated cells was significantly increased on day 1 after stress. On day 10 after stress, total number of activated cells was significantly increased while the proportion of GAD67^+^ type cells in the activated cells returned to the control levels. Additionally, the proportion of activated cells in the CaMKII^+^ type cells was also significantly increased in BLA.

**FIGURE 4:**
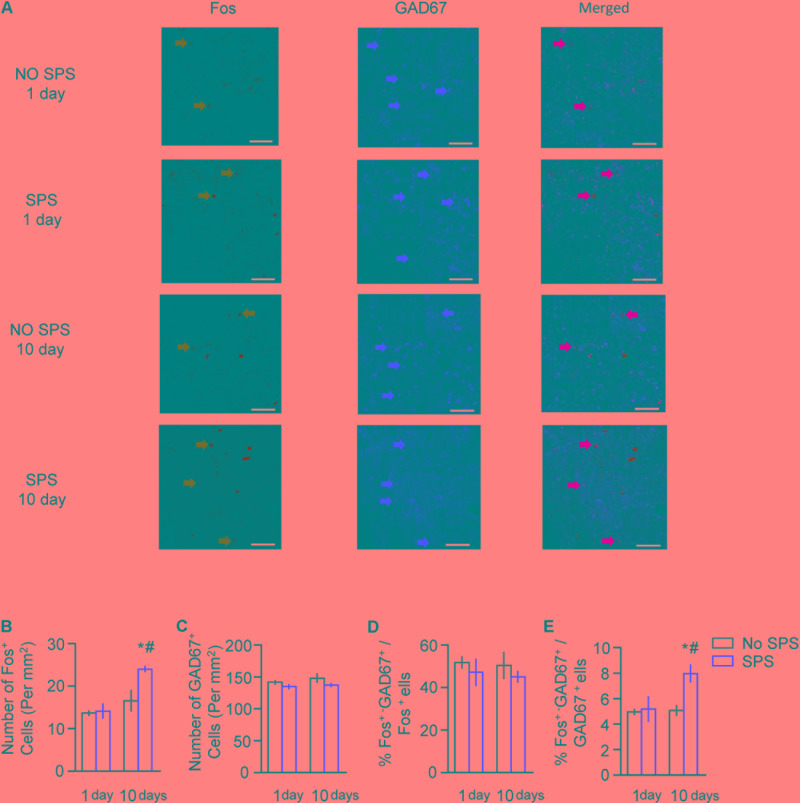
Effect of single prolonged stress (SPS) on activation of GABAergic neurons in CeA on day 1 or day 10 after stress. **(A)** Representative images showing **green** (Fos protein), red GAD67 protein), and merged channels of double-label neurons in CeA in different experimental conditions. Scale bars represent 100 μm. **(B,C)** Effect of the experimental manipulations on Fos protein **(B)** and GAD67 expression **(C)** in CeA. **(D)** Percentage of GABAergic neurons in activated cells of CeA in different experimental manipulations, as calculated by number of overlap (Fos + GAD67 protein-IR)/Fos-IR. **(E)** Percentage of activated cells in GABAergic neurons of CeA in different experimental manipulations, as calculated by number of overlap (Fos + GAD67 protein-IR)/GAD67-IR. *n* = 7–8 per group. Data are mean ± SEM. ^∗^Different from no SPS group at each post-stress day. ^#^Different from SPS-1 day group, one-way ANOVA, *p* < 0.05.

For immunofluorescence data of Fos and GAD67 in CeA (**Figure 4A**), the analysis revealed significant effects of Stress (*F*_1,27_ = 6.40, *p* < 0.05 for number of Fos^+^ cells; *F*_1,27_ = 5.17, *p* < 0.05 for percentage of Fos^+^-GAD67^+^/GAD67^+^) and Post-stress Day (*F*_1,27_ = 16.73, *p* < 0.01 for number of Fos^+^ cells; *F*_1,27_ = 4.46, *p* < 0.05 for percentage of Fos^+^-GAD67^+^/GAD67^+^). *Post hoc* analysis revealed a significant increase in number of Fos^+^ cells and Fos^+^- GAD67^+^/GAD67^+^ on day 10 (both *p* < 0.05, **Figures 4B,E**). There are no significant changes of the total number of the GAD67^+^ cells and the percentage of Fos^+^-GAD67^+^/Fos^+^ after stress on day 1 and day 10 (both *p* > 0.05, **Figures 4C,D**). These results suggested that the numbers of activated cells and GAD67^+^ type neurons as well as cell types in the activated cells were not affected on day 1 after stress. However, on day 10 after stress, total number of activated neurons and the proportion of activated cells in the GAD67^+^ type cells were significantly increased in CeA.

These results indicate that SPS produces distinct pattern of neuronal activation in BLA and CeA on day 1 and day 10 after stress, which is consistent with previous study that the PTSD-associated behaviors accompanied with dysfunction of glutamatergic and GABAergic transmission in central nervous system (Knox et al., 2010).

## Discussion

Post-traumatic stress disorder (PTSD) is a chronic and debilitating psychiatric disorder caused by experience of TEs. PTSD is characterized by the intrusive re-experiencing of past trauma, avoidance behavior, enhanced fear, and hyperarousal following a TE in the vulnerable populations (APA, 2013a). In present study, we used the SPS procedure to explore the underlying circuitry mechanism of PTSD. We found that PTSD-associated behaviors occurred on day 10, not day 1 after SPS. The neuronal activity in amygdala displayed distinct pattern at different time after stress. On day 1 after stress, we observed a reduction of the proportion of activated glutamatergic neurons but an increase of the proportion of activated GABAergic neurons in BLA. On day 10 after stress, there were increases of both activated glutamatergic and GABAergic neurons in BLA, and an increase of activated GABAergic neurons in CeA. These results indicate that during early and late phase after traumatic stress, distinct patterns of activation of glutamatergic neurons and GABAergic neurons in amygdala may be involved in the development of PTSD.

After the SPS procedure, we tested the behavioral changes of the SPS-rats compared with that of the unstressed-controls, using the behavioral tests of OFT, EPM, and CFC. Our data revealed that there were normal anxiety-like behaviors and fear extinction 1 day after SPS, but an enhancement of anxiety-like behaviors and an impairment of memory retention of fear extinction 10 days after SPS comparing to the unstressed-controls. In previous studies, the SPS-7 days group showed an enhanced cued freezing compare to controls, which indicated an extinction retention deficit in the SPS-7 days group but not in the SPS-1 day group (Knox et al., 2012). Consistent with this, the quiescent period is thought to be essential for the development of PTSD-like behaviors (Liberzon et al., 1999). This phenomenon was considered to be in relation to the specific roles of high-level glucocorticoid receptors versus downregulated mineralocorticoid receptors on HPA axis in hippocampus up to 14 days after SPS (Liberzon et al., 1997, 1999). In our study, we found a distinct pattern of glutamatergic and GABAergic neuronal activations in BLA and CeA in early and late phase after stress and suggested that dysfunction of excitatory/inhibitory balance maybe a new mechanism underlying quiescent period of PTSD-associated behaviors.

Strongly involved in forming emotional memories, especially fear-related memories, the amygdala is thus regarded as the central brain region and attracts many scientists’ special attention. Using animal model of PTSD, previous studies have found that glucocorticoid receptor (Kohda et al., 2007), 5-HT_2_C (Harada et al., 2008), Rin1 and Stathmin (Han et al., 2017), beta-adrenoreceptors (Ronzoni et al., 2016), and beta-arrestin-2 (Ding et al., 2017) are involved in the PTSD-associated behaviors. Besides, the amygdala is composed of several functionally distinct nuclei that interact during stress responses. In the amygdala, the BLA is in receipt of early multimodal sensory information from the thalamus and cortex, and thus is considered as the major input station (Höfner, 1991; Mcdonald, 1998). The CeA projection neurons could contact different structures in the brainstem or hypothalamus to orchestrate conditioned autonomic and motor responses (Krettek and Price, 1978; Veening et al., 1984; Ledoux et al., 1988; Mcdonald, 1991; Petrovich and Swanson, 1997; Royer et al., 1999). The other findings indicate amygdala nuclei connected to many other cortical and subcortical structures reciprocally or unidirectionally, and participate different of behaviorally relevant outputs (Mcdonald, 1991, 1998; Mcdonald et al., 1996; Pitkänen et al., 2000). Some evidence suggests that the two main sub-regions of amygdala may play different roles in emotional regulation. For example, it was showed that the reciprocal projections between BLA and CeA and medial prefrontal cortex (mPFC) are different, and the transmissions from BLA but not CeA to mPFC play a critical role in fear memory and extinction (Sah et al., 2003; Davis et al., 2017; Klavir et al., 2017). Thus, we analyzed the molecular changes in BLA and CeA, respectively. We found for BLA, its activation was slightly decreased on day 1, and increased on day 10. The activation of CeA was not affected on day 1, and was increased on day 10. Our study suggests that during early and late phase after traumatic stress, distinct patterns of activation of glutamatergic neurons and GABAergic neurons are displayed in BLA and CeA and may provide clues for when and which prefrontal regions are selected for neuromodulation treatment in PTSD patients. In the future, more studies are needed to investigate whether subregions of CeA, such as CEl and CEm, could play different roles in the behavioral changes after traumatic stress. In addition, the information can be processed by not only the amygdala networks but also interactions with other brain structures (Pitkänen et al., 1997; Sah et al., 2003; Ehrlich et al., 2009). For example, descending inhibitory inputs from the mPFC regulate the transmission of excitatory neurotransmission from the BLA to the CeA, which plays a role in the extinction of conditioned fear responses (Likhtik et al., 2008; Peters et al., 2009). Furthermore, decreases in the activity of excitatory neurons in the mPFC were found in SPS-rats (Knox et al., 2010). Thus, further more studies should investigate how amygdala interacts with other brain regions to regulate the development of PTSD-like behaviors.

It was showed that exposure to stress can change GABAergic ability and disrupt the inhibition/excitation balance in emotion-related circuits in the brain (Ritov et al., 2015; Ardi et al., 2016). The glutamatergic excitatory and GABAergic inhibitory neurons also have well established roles in the regulation of expression and extinction of fear memory (Zhang and Cranney, 2008; Bolshakov, 2009; Makkar et al., 2010; Pape and Pare, 2010; Shankar and Minz, 2010). However, few lines of evidence suggested the roles of GABAergic and glutamatergic systems of amygdala in the neuropathology of PTSD. In our study, a reduction in the numbers of CaMKII positive cell was observed after the traumatic stress. There are two possible explanations for the results. One is that the CaMKII levels were decreased in glutamatergic neurons and another one is that the total number of glutamatergic neurons was decreased. Both of these situations could result in decreased CaMKII protein levels in BLA, which is consistent with a previous study in which the CaMKII was examined using western blot and a decreased CaMKII level in BLA was observed on day 1 after SPS (Xiao et al., 2009). Furthermore, although the total levels of CaMKII were decreased in BLA, the percentage of Fos^+^-CaMKII^+^/CaMKII^+^ was increased, suggesting that traumatic stress reorganized neural ensembles. Previous studies suggest that different neural populations in amygdala may play different roles in anxiety-like behaviors, emotional memory formation, and memory extinction (Meins et al., 2010; Marek et al., 2013; Bukalo et al., 2015). Furthermore, CaMKII in amygdala is implicated in both memory formation and extinction (Rodrigues et al., 2004; Alvarez-Dieppa et al., 2016; Tran and Keele, 2016). Thus, we speculate that traumatic stress may produce different effects on CaMKII levels in different neuronal ensembles and contribute to different PTSD-associated behaviors, such as anxiety and deficit in fear memory extinction.

In addition, we found that the proportion of activated glutamatergic was reduced in BLA on day 1 after traumatic stress. Activated glutamatergic and GABAergic neurons were both increased in BLA and activated GABAergic neurons was increased in CeA on day 10 after stress. These results indicated that the hyperactivity of glutamatergic neurons or the imbalance of excitatory/inhibitory tune in BLA and CeA may be implicated in the development of anxiety-like behaviors. Our results were consistent with previous study that the activation of inhibition in BLA was increased in the resilience phenotype, and activations of BLA and CeA were both increased in rats with PTSD-like behaviors (Ritov et al., 2015, 2016). However, there are other cell types in amygdala, such as corticotrophin releasing factor neurons, which also play important roles in fear and anxiety behaviors (Gafford and Ressler, 2015). Future studies should investigate other cell types as well as the interactions between these cell types and glutamatergic and GABAergic neurons in amygdala during the development of PTSD-associated behaviors.

In the past two decades, critical advances have been made in the balance of excitation and inhibition. This balance governs the memory, learning, attention, intelligence, and thinking, involving the dominant aspects of cognitive function. Excitation/inhibition imbalance was demonstrated to related with a variety of disorders, such as drug abuse (den Boon et al., 2015), autism spectrum disorder (Dickinson et al., 2016; Smith et al., 2016), Alzheimer’s disease (Povysheva and Johnson, 2016), schizophrenia (Lisman, 2012), Down syndrome (Souchet et al., 2014), epileptic syndromes (Petroff et al., 1996), Tourette’s syndrome (Singer and Minzer, 2003), certified by the pharmacological, transgenic, and electrophysiological technology in these articles. And existing studies have been concentrated at the brain regions, involving the PFC, hippocampus, cerebellum, and occipital lobe (Petroff et al., 1996; Lisman, 2012; Souchet et al., 2014; den Boon et al., 2015; Povysheva and Johnson, 2016; Krystal et al., 2017). However, this similar research on the neurobiological mechanisms of PTSD, especially in amygdala, is not fully clear. Our study was the first to explore the contribution of the balance of excitation and inhibition to PTSD associated behaviors. However, we only showed the correlation between the behavioral changes and the activity of both excitation and inhibition neurons after traumatic stress. Future studies should combine electrophysiology, optogenetic or transgenic technologies to elucidate the causal role of excitation/inhibition in amygdala for drug development of PTSD.

## Author Contributions

QF, JS, J-LY, and LL designed the study. QF, ZL, G-DH, H-HZ, Y-YC, and L-BZ performed the study. QF, ZL, and Z-BD analyzed the results and wrote the paper together. All the authors have read and approved the final version of the manuscript.

## Conflict of Interest Statement

The authors declare that the research was conducted in the absence of any commercial or financial relationships that could be construed as a potential conflict of interest.
